# Anatomy of the smooth muscle structure in the female anorectal anterior wall: convergence and anterior extension of the internal anal sphincter and longitudinal muscle

**DOI:** 10.1111/codi.14549

**Published:** 2019-01-24

**Authors:** S. Muro, Y. Tsukada, M. Harada, M. Ito, K. Akita

**Affiliations:** ^1^ Department of Clinical Anatomy Tokyo Medical and Dental University Tokyo Japan; ^2^ Department of Colorectal Surgery National Cancer Center Hospital East Chiba Japan

**Keywords:** Rectum, anal canal, vagina, perineal body, internal anal sphincter, longitudinal muscle

## Abstract

**Aim:**

The anatomy of the region between the vagina and anal canal plays an essential role when performing a proctectomy for low‐lying tumours. However, the anatomical characteristics of this area remain unclear. The purpose of the present study was to clarify the configuration, and both lateral and inferior extensions, of the muscle bundles in the anorectal anterior wall in females.

**Methods:**

Using cadaveric specimens, macroscopic anatomical and histological evaluations were conducted at the anatomy department of our institute. Macroscopic anatomical specimens were obtained from six female cadavers. Histological specimens were obtained from eight female cadavers.

**Results:**

The smooth muscle fibres of the internal anal sphincter and longitudinal muscle extended anteriorly in the anorectal anterior wall of females and the muscle bundles showed a convergent structure. The anterior extending smooth muscle fibres merged into the vaginal smooth muscle layer, distributed subcutaneously in the vaginal vestibule and perineum and spread to cover the anterior surface of the external anal sphincter and the levator ani muscle. Relatively sparse space was observed in the region anterolateral to the rectum on histological analysis.

**Conclusion:**

Smooth muscle fibres of the rectum and vagina are intermingled in the median plane, and there is relatively sparse space in the region anterolateral to the rectum. Therefore, when detaching the anorectal canal from the vagina during proctectomy, an approach from both the lateral sides should be used.

**What does this paper add to the literature?**

This study reports the detailed anatomy of the anterior extending smooth muscle fibres of the anorectal canal, which has not been previously studied. We propose an ideal approach when detaching the anorectal canal from the vagina during proctectomy on the basis of the findings of a precise histological analysis.

## Introduction

Surgery for lower rectal tumours (e.g. intersphincteric resection or transanal total mesorectum excision) has evolved remarkably in recent years 1, 2, 3, 4, leading to an increase in the demand for more detailed anatomical definition of the anorectal canal.

Although the basic structure comprises the circular muscle (internal anal sphincter), longitudinal muscle and external anal sphincter, the anorectal muscle layer has several complex elements that make it difficult to comprehend. For example, the muscle bundle of the levator ani muscle inserts between the longitudinal muscle and external anal sphincter 5, and the longitudinal muscle penetrates the subcutaneous part of the external anal sphincter muscle 6. These structures make the anorectal muscular layer complicated. The muscle layer of the anorectal anterior wall in males is particularly complex. One of the complicating factors is the anterior extension of the smooth muscle layer, and it is known that several fibres of the longitudinal muscle extend anteriorly to form the rectourethralis muscle in males 7, 8, 9, 10, 11, 12.

In females, two simple layers of smooth muscle are thought to be located in the anorectal anterior wall, and a fibromuscular mass, called the perineal body, is believed to exist between the anorectal canal and vagina 13, 14, 15. However, given the homology of male and female structures, the existence of a similar smooth muscle extension structure is inferred in the anorectal anterior wall. In fact several reports have described internal anal sphincter and longitudinal muscle fibres extending anteriorly into the anorectal anterior wall of females 16, 17, 18. However, the anatomical details remain unclear, in particular the muscle bundle configuration of the anteriorly extending muscles as well as the spread of the smooth muscle tissue.

During the transanal approach for rectal tumours, the rectourethralis muscle of males can be clearly visualized under a magnified view, and it is recognized as an important structure when dissecting the anterior rectal wall 19. If a structure homologous to the rectourethralis muscle in males is found in females, and if its anatomical details can be clarified, these results would present important information for the transanal approach and assist in the selection of the optimal dissection line during surgery.

We hypothesized that part of the smooth muscle layer of the anorectal canal extends anteriorly and spreads laterally and inferiorly in females and that the accompanying changes in the smooth muscle bundle are manifested in the anterior anorectal wall. The purpose of the present study was to clarify the configuration of the muscle bundles in the anterior anorectal wall and the anterior extension structure of the smooth muscle, including the lateral and inferior extent, in females.

## Method

### Cadavers

All cadavers used in this study were obtained from the dissecting room of our institute. The cadavers were donated to the Department of Anatomy at our institute for use in clinical studies within the guidelines of the Act on Body Donation for Medical and Dental Education law in Japan. The cadavers were fixed by arterial perfusion with 8% formalin and preserved in 30% alcohol to prevent fungal growth and maintain tissue softness.

### Macroscopic anatomy

Six female cadavers (age range 42–80 years; mean age 65.7 years) were used for dissection. Each pelvis, including the rectum, anus, adjacent muscular tissues and connective tissues, was obtained *en bloc* from the cadavers. Three pelvises were sectioned in the sagittal plane and dissected from the medial aspect. The remaining three pelvises were sectioned in the coronal plane through the anorectal canal and dissected from the posterior aspect. The anorectal canal was dissected from the luminal side to demonstrate the circular muscle and longitudinal muscle.

### Histology

Histological analysis was performed using female pelvises (eight bodies; 14 sides; age range 66–96 years; mean age 84.5 years). Pelvises were obtained *en bloc* and sectioned in the sagittal plane. In the 12 hemi‐pelvises, the tissue of the region anterior to the anorectal canal was obtained from the section to create a histological specimen of the sagittal plane. The obtained tissue was embedded in paraffin and sectioned into specimens 5‐μm thick. In the other two hemi‐pelvises, the tissue in the region anterior to the anorectal canal was obtained *en bloc* to create a histological specimen from the transverse plane. The tissue was embedded in paraffin and serially sectioned into specimens 5‐μm thick at intervals of 1 mm.

The histological sections were stained with elastic Van Gieson (EVG). In addition, to confirm the distribution of smooth muscle and skeletal muscle tissues, immunohistological staining was performed with anti‐smooth actin (ready‐to‐use Actin, Smooth Muscle Ab‐1, Clone 1A4; Thermo Fisher Scientific, Fremont, California, USA) and anti‐skeletal myosin (ready‐to‐use Myosin, Skeletal Muscle Ab‐2, Clone MYSN02; Thermo Fisher Scientific).

### Three‐dimensional reconstruction

The internal anal sphincter, longitudinal muscle, external anal sphincter, levator ani and vaginal muscularis were analysed using computer‐assisted three‐dimensional reconstruction. Three‐dimensional reconstructions were made from histological serial transverse sections of a female specimen (66 years old at death). All of the serial sections were scanned and the muscles were traced and coloured. Section sequences were reconstructed using SrfII software (SrfII, Ratoc, Tokyo, Japan).

### Ethical approval

Study approval was obtained from the Board of Ethics of our institute (M2018‐006).

## Results

**Figure 1:**
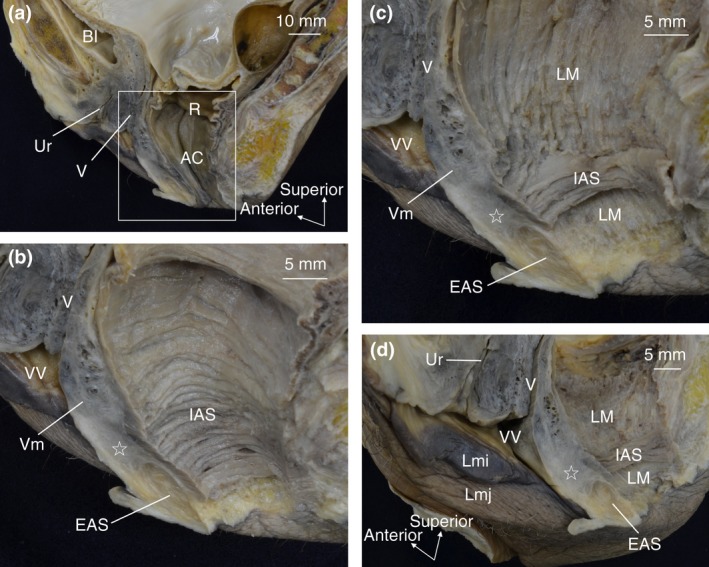
A female hemi‐pelvis observed from the sagittal section. The woman was 66 years old at death and had a history of giving birth. The internal anal sphincter and longitudinal muscle are shown according to layers. Their muscle bundles converged into the anal anterior wall (indicated by the star). AC, anal canal; Bl, bladder; EAS, external anal sphincter; IAS, internal anal sphincter; LM, longitudinal muscle; Lmi, labium minus; Lmj, labium majus; R, rectum; Ur, urethra; V, vagina; Vm, vaginal muscularis (muscle layer of vagina); VV, vaginal vestibule. ☆, The region where IAS and LM converge and extend anteriorly.

The anorectal canal, vagina and urethra were observed in the sagittal section of female pelvises (Fig. 1a). The mucous membrane was removed to demonstrate the circular muscle layer (internal anal sphincter; Fig. 1b). Subsequently, the circular muscle was gradually removed to demonstrate the longitudinal muscle. The muscle bundles of the internal anal sphincter and longitudinal muscle converged into the anal anterior wall (indicated by the star in Fig. 1c). The anterior aspect of this region (indicated by the star in Fig. 1c, d) corresponded to the span from the inferior end of the vagina to the vaginal vestibule and the area posterior to it (Fig. 1d).

**Figure 2:**
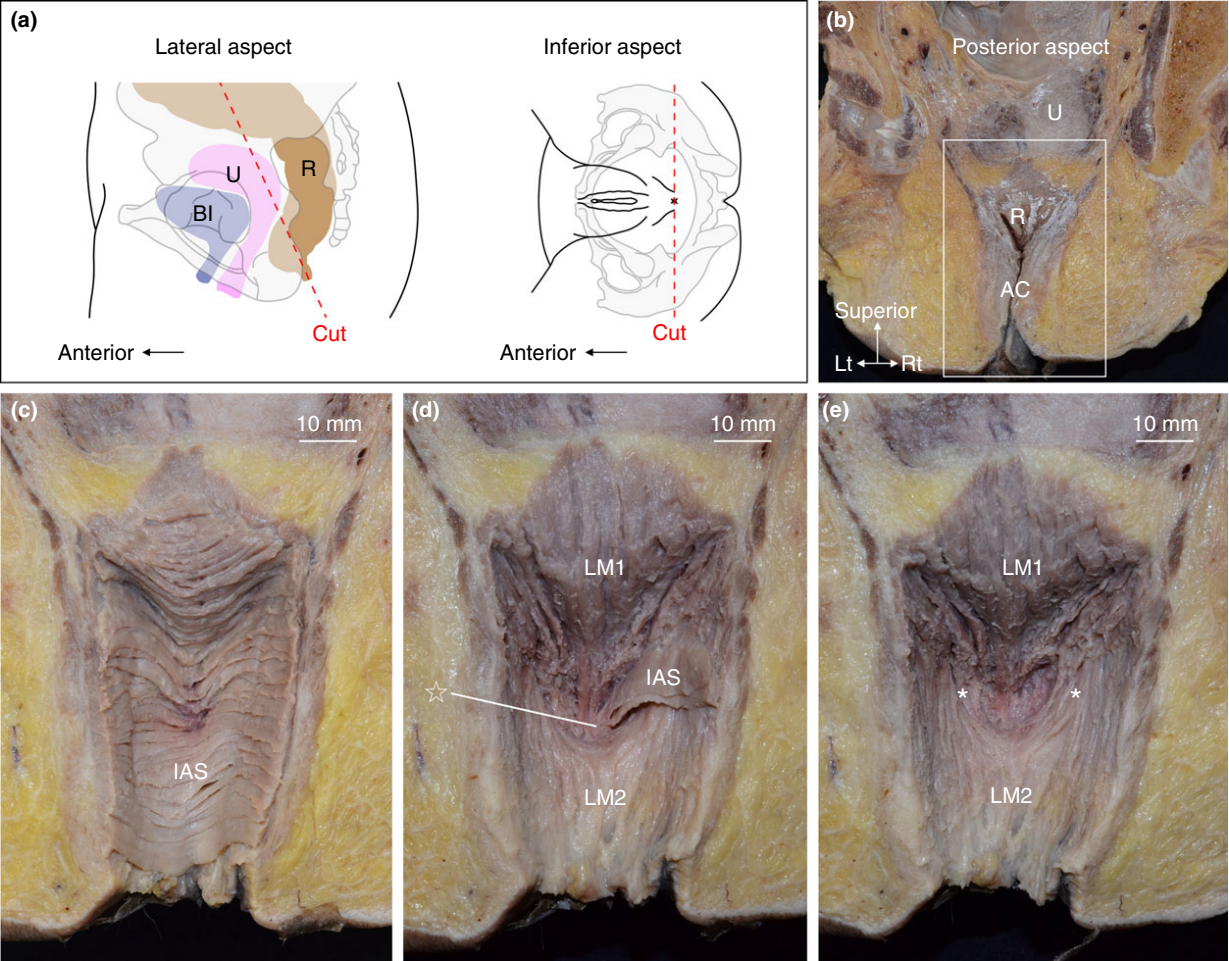
Anorectal anterior wall observed from the luminal side (from the posterior aspect). The woman was 79 years old at death and had a history of giving birth. The muscle bundles of the internal anal sphincter and longitudinal muscle converged to the median region and extended anteriorly (indicated by the star). The longitudinal muscle bundles descending from the median (LM1) converged and anteriorly extended, whereas the bundles descending more laterally (indicated by the asterisks) curved to the midline were adjoined to each other and descended (LM2). IAS, internal anal sphincter; LM, longitudinal muscle. ☆, The region where IAS and LM converge and extend anteriorly; *, longitudinal muscle bundles descending on the anterolateral wall of the anorectal canal.

The anorectal anterior wall was dissected from the luminal side (posterior side) of the pelvises in which the posterior part of the anorectal canal was removed (Fig. 2a, b). The mucous membrane was removed to demonstrate the circular muscle layer (internal anal sphincter; Fig. 2c). Subsequently, the circular muscle was gradually removed to demonstrate the longitudinal muscle. The muscle bundles of the internal anal sphincter and longitudinal muscle converged in the median region and extended anteriorly (indicated by the star in Fig. 2d). The converging muscle bundles of the longitudinal muscle were those that descended from the median and paramedian regions (indicated by LM1 in Fig. 2d, e). The muscle bundles descending more laterally (indicated by the asterisk in Fig. 2e), gradually curving to the midline, were adjoined to each other and descended in parallel (indicated by LM2 in Fig. 2d, e). As a result, the longitudinal muscle bundle in the median anterior wall was not continuous between the rectal level (LM1) and anal level (LM2).

**Figure 3:**
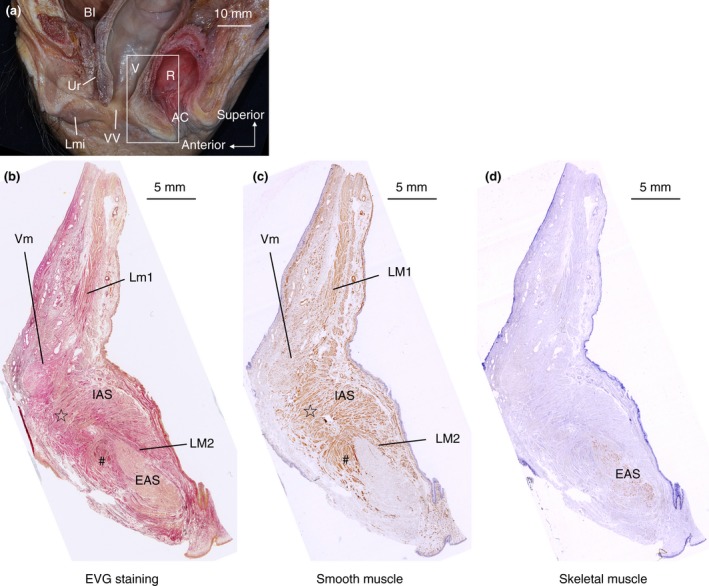
The sagittal section of the anorectal anterior wall stained with elastic Van Gieson and immunostaining. The woman was 87 years old at death and had no history of giving birth. The smooth muscle fibres of the internal anal sphincter and rectal longitudinal muscle extended anteriorly (indicated by the star). The smooth muscle fibres of the rectal longitudinal muscle intermingled with the smooth muscle of the vagina. The smooth muscle fibres extended from the anal longitudinal muscle and covered the anterior surface of the external anal sphincter (indicated by the number sign). AC, anal canal; Bl, bladder; EAS, external anal sphincter; IAS, internal anal sphincter; LM, longitudinal muscle; Lmi, labium minus; R, rectum; Ur, urethra; V, vagina; Vm, vaginal muscularis (muscle layer of vagina); VV, vaginal vestibule. ☆, The region where the IAS and LM converge and extend anteriorly; #, smooth muscle tissue located in front of the EAS.

The sagittal section of the anorectal anterior wall was examined histologically (Fig. 3). EVG staining demonstrated that part of the internal anal sphincter protruded anteriorly in the anal anterior wall (indicated by the star in Fig. 3b). On the superior and inferior sides of the protruding internal anal sphincter, the fibres of the longitudinal muscle also extended anteriorly. Immunostaining confirmed that the anterior extending fibres included not only collagen fibres but also abundant smooth muscle fibres (Fig. 3c). The longitudinal muscle of the rectum (indicated by LM1 in Fig. 3c) extended anteriorly and intermingled with the smooth muscle layer of the lower posterior vaginal wall. Therefore, the rectum and lower vagina had no clear boundaries at this level (Fig. 3c). However, the longitudinal muscle of the anal canal (indicated by LM2 in Fig. 3c) extended and covered the anterior surface of the external anal sphincter (indicated by the number sign in Fig. 3c). As a result, the rectal longitudinal muscle (LM1) and the anal longitudinal muscle (LM2) were not continuous in the median plane (Fig. 3b, c). The muscle fibres of the external anal sphincter, detected by anti‐skeletal muscle antibody, were localized, whereas the smooth muscle tissue was distributed more widely (Fig. 3c, d). The anterior extending smooth muscle fibres spread to the smooth muscle layer of the lower vagina, the subcutaneous region of the vaginal vestibule and the perineum, and anterior to the external anal sphincter (Fig. 3c). Consequently, the region anterior to the anorectal canal was occupied by smooth muscle tissue.

**Figure 4:**
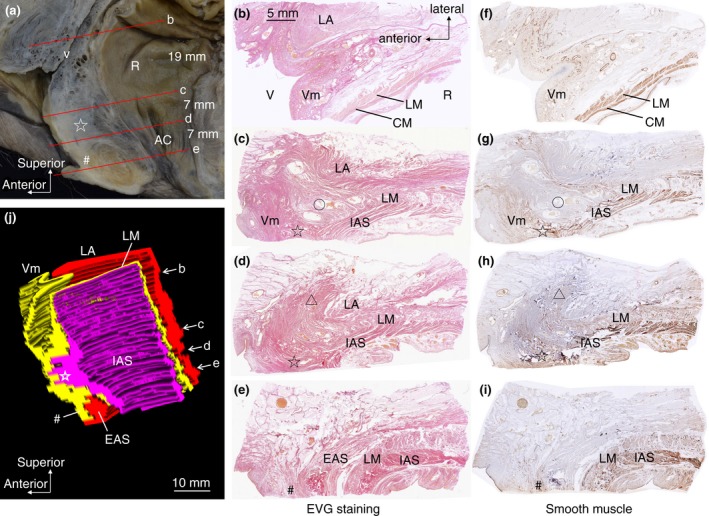
The transverse section of the anorectal anterior wall stained with elastic Van Gieson and immunostaining. Three‐dimensional reconstruction was performed from serial sections. The woman was 66 years old at death and had a history of giving birth. The smooth muscle fibres of the internal anal sphincter and the longitudinal muscle anteriorly extended and intermingled with the smooth muscle of the vagina in the median (indicated by the star). Lateral to that, a relatively sparse space was observed (indicated by the circle). Several smooth muscle fibres were observed on the anterior surface of the levator ani and the external anal sphincter (indicated by the triangle and the number sign). AC, anal canal; Bl, bladder; EAS, external anal sphincter; IAS, internal anal sphincter; LM, longitudinal muscle; R, rectum; V, vagina; Vm, vaginal muscularis (muscle layer of vagina); VV, vaginal vestibule. ☆, The region where the IAS and LM converge and extend anteriorly; *, longitudinal muscle bundles descending on the anterolateral wall of the anorectal canal; #, smooth muscle tissue located in front of the EAS; ▵, region where smooth muscle tissue extends laterally; ○, relatively sparse space in the anterolateral to the anorectal canal.

Figure 4 shows transverse sections at several levels and a three‐dimensional reconstruction. At the superior level, rough connective tissue was present between the rectum and vagina, and the rectal wall and vaginal wall were clearly distinguishable (Fig. 4b, f). Around the superior border of the anal canal, the smooth muscle fibres of the anterior longitudinal muscle extended and intermingled with the smooth muscle layer of the vagina in the median region (Fig. 4c, g). However, in the region anterolateral to the rectum, there was a relatively sparse space where vessels and nerves passed (indicated by the circle in Fig. 4c, g). In the anterior wall of the anal canal, the smooth muscle fibres of the internal anal sphincter and longitudinal muscle extended anteriorly, spread laterally and covered the anterior side of the levator ani muscle (indicated by the triangle in Fig. 4d, h). At the inferior level, the layered structures of the internal anal sphincter, longitudinal muscle and external anal sphincter were confirmed (Fig. 4e, i). Several smooth muscle fibres were observed anterior to the external anal sphincter (indicated by the number sign in Fig. 4e, i).

## Discussion

**Figure 5:**
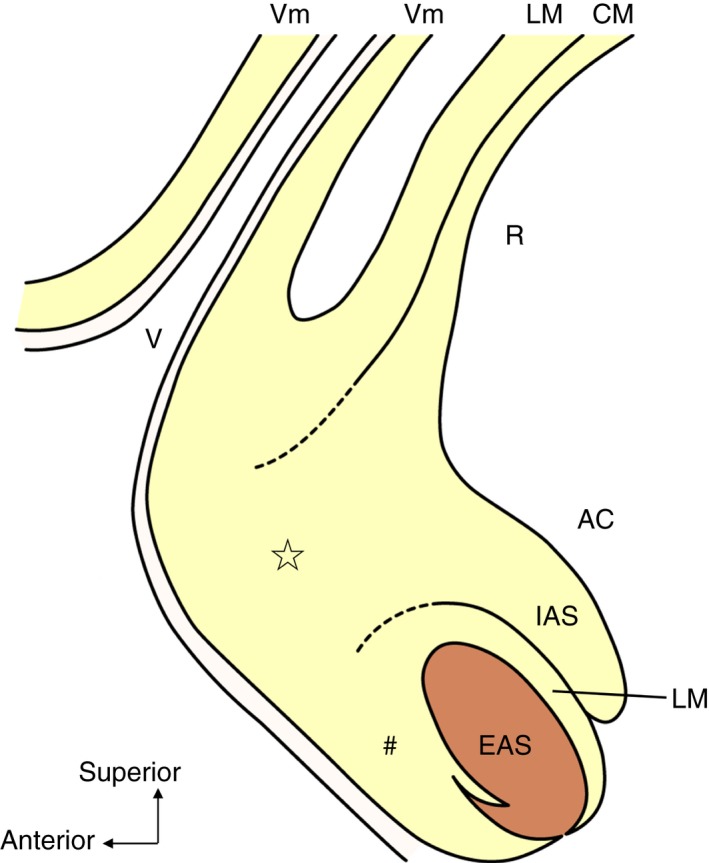
Anterior extension of the smooth muscles in the region anterior to the anorectal canal in females. The smooth muscle fibres of the circular muscle extend anteriorly and are distributed subcutaneously. The smooth muscle fibres of the rectal longitudinal muscle intermingle with the muscle layer of the vagina. The smooth muscle fibres of the anal longitudinal muscle cover the external anal sphincter. AC, anal canal; EAS, external anal sphincter; IAS, internal anal sphincter; LM, longitudinal muscle; R, rectum; V, vagina; Vm, vaginal muscularis (muscle layer of vagina). ☆, The region where the IAS and LM converge and extend anteriorly; #, smooth muscle tissue located in front of the EAS.

The present study confirmed that the smooth muscle fibres of the internal anal sphincter and longitudinal muscle extended anteriorly in the anterior wall of the anorectum of females and clarified that the smooth muscle fibres spread laterally and inferiorly. It also revealed that the muscle bundles exhibit a characteristic convergent structure. The anterior extending smooth muscle fibres merged into the vaginal smooth muscle layer, distributed subcutaneously in the vaginal vestibule and perineum, and spread to cover the anterior surface of the external anal sphincter and levator ani muscle (Fig. 5).

**Figure 6:**
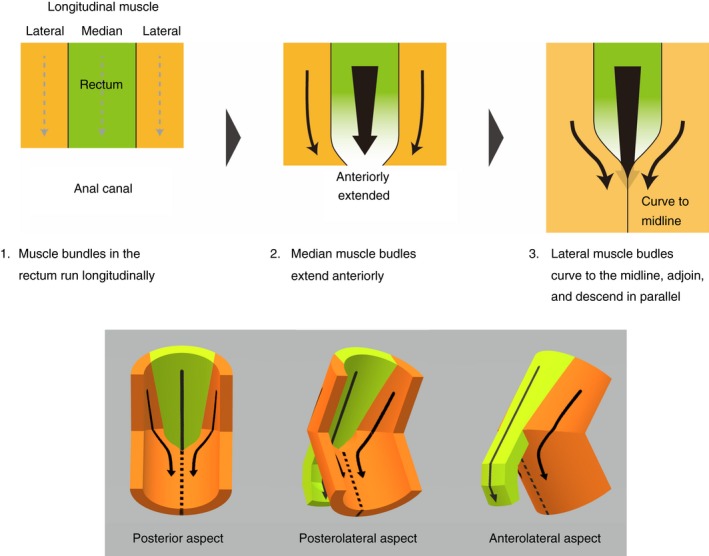
Morphological model of the longitudinal muscle bundle in the anorectal anterior wall (seen from the luminal side or posterior aspect). The median longitudinal muscle bundles extended anteriorly, and the lateral bundles curved to the midline and were adjoined to each other.

Oh and Kark 16 described an anterior extension of several fibres of the rectal longitudinal muscle together with the internal anal sphincter in females; they termed these muscle fibres collectively as the rectovaginalis muscle, and considered this the structure in females that corresponded to the rectourethralis muscle in males. Aigner *et al*. 17 reported additional longitudinal muscle bundles within the rectogenital septum, using foetal and newborn specimens. Kinugasa *et al*. 18 also reported that part of the rectal longitudinal muscle of females extended anteriorly toward the vagina and vaginal vestibule; they termed it the anterior conjoint longitudinal coat. Our study confirmed that the smooth muscle fibres of the internal anal sphincter and longitudinal muscle extend anteriorly in the anorectal anterior wall of females (Fig. 5). Furthermore, our study clarified the course of smooth muscle bundles and distribution of smooth muscle tissue, including not only the anterior extension but also the lateral extent. Of particular note is the characteristic course of the muscle bundles, which is associated with anterior protrusion of the internal anal sphincter and longitudinal muscle. The muscle bundles of the internal anal sphincter and longitudinal muscle showed a convergent course toward the anterior anal wall. In addition, the longitudinal muscle in the median plane was not continuous between the rectal and anal levels as the median longitudinal muscle bundles extended anteriorly whilst the lateral bundles curved to the midline and were adjoined to each other (Fig. 6).

The anterior extension of the smooth muscle of the anorectal canal has been confirmed in males. The smooth muscle structure anteriorly, extending to the posterior urethra, is termed the rectourethralis muscle, whereas the smooth muscle structure extending antero‐inferiorly to cover the anterior surface of the external anal sphincter was reported as the anterior bundle of the longitudinal muscle 7, 8, 9, 10, 11, 12. The anterior extension structure of the internal anal sphincter and rectal longitudinal muscle described in our study (indicated by the star in Figs 3b, c and 4d, h) is considered to be the structure homologous to the rectourethralis muscle of males, on the basis of the location, morphology and tissue composition. However, the smooth muscle fibres covering the anterior surface of the external anal sphincter (indicated by the number sign in Figs 3b, c and 4f, i) seem to correspond to the anterior bundle of the longitudinal muscle in males. This structure in females is probably composed of muscle bundles that differ from those in males. Although the anterior bundle of the longitudinal muscle of males is composed of the directly descending longitudinal muscle bundles of the rectum, the corresponding structure in females is composed of the longitudinal muscle bundles curving anterolaterally to the midline. This difference is important, considering the morphological differences of the perineum between males and females.

The term ‘perineal body’ is often used to describe the anatomy of the pelvic floor and perineal region. Additionally, researchers who clarified the anatomical planes for an extralevator abdominoperineal excision have for a long time realized that the interface between the anterior anorectal wall and the structures of the posterior genitourinary complex have many similarities in males and females, including a fibromuscular point (perineal body) that serves as a connecting point in the middle of the non‐bony pelvis 14, 17. The perineal body refers to the fibromuscular tissue in the region between the rectum and urogenital structures 10, 12, 16. Larson *et al*. 20 reported in detail the muscles that compose the perineal body in their MRI study. The anterior extension of the internal anal sphincter and longitudinal muscle shown in the present study can be interpreted as one of the components of the female perineal body.

The rectourethralis muscle in males is thought to contribute to the stabilization of the membranous urethra and to be a determinant of anorectal flexure, given that it connects the rectal wall to the membranous urethra 9, 21, 22, 23. The similar smooth muscle extension in females is characteristic of merging with the smooth muscle of the vaginal posterior wall and covering the anterior surface of the external anal sphincter. The external anal sphincter is covered on the lumen side by the longitudinal muscle; therefore, the external anal sphincter in the anterior anal wall is surrounded by smooth muscle tissue on the anterior and posterior sides. On the basis of such morphological features, we postulate that the smooth muscle extension of the anorectal anterior wall of females contributes to stabilization of the lower part of the posterior vaginal wall and the external anal sphincter of the anterior wall of the anal canal.

In recent years, surgery for lower rectal tumours (e.g. intersphincteric resection or transanal total mesorectum excision) has evolved remarkably 1, 2, 3, 4. In addition, the rectourethralis muscle of males has attracted much attention as an important structure when dissecting the anorectal anterior wall 19. Our study identified the structure in females that is homologous to the rectourethralis muscle in males and clarified its anatomical details. When performing a surgical resection along the external surface of the longitudinal muscle, such as in an intersphincteric resection, it is necessary to recognize that the internal anal sphincter and longitudinal muscle extend anteriorly; furthermore, it is impossible to proceed with dissection without cutting the anteriorly protruding smooth muscle fibres. Because the rectal longitudinal muscle and vaginal muscularis have intermingling smooth muscle fibres in the median region, a definite release layer cannot be easily found in the midline. However, there is a relatively sparse space in the region anterolateral to the rectum. Therefore, it would be preferable to approach from both lateral sides when detaching the anorectal canal from the vagina. In the more distal region, this sparse space becomes smaller and eventually disappears, making it difficult to detach the anorectum from the vagina.

The smooth muscle structure revealed in our study may also be incriminated in the pathology of pelvic organ prolapse. Kinugasa *et al*. 18 reported that smooth muscle fibres extending anteriorly from the anorectal canal may work to avoid vaginal prolapse. Boreham *et al*. 24 reported that, for women with posterior vaginal prolapse, the smooth muscle bundles in the posterior vagina were disorganized and decreased and nerve bundles and ganglia were also reduced. Our study demonstrated that the structure in which the rectal smooth muscle anteriorly extended and merged into the vaginal smooth muscle layer seemed to contribute to the stabilization of the lower part of the vaginal posterior wall and is probably an important structure for preventing vaginal prolapse.

In general, it is difficult to acquire young, nulliparous cadavers; hence, gravid cadavers were included in this study. Therefore, changes in the fibromuscular architecture associated with childbirth, including occult damage to smooth muscle, cannot be ruled out. For future research, a study comparing female anatomy with and without childbirth history, or a study using foetal specimens, may give additional information.

## Conclusion

The present study confirmed that part of the anorectal smooth muscle layer extends anteriorly from the anorectal anterior wall of females, thus clarifying the lateral extent of the smooth muscle tissue and characteristic convergent structure of the muscle bundles of the internal anal sphincter and longitudinal muscle. Our anatomical findings indicated that, when dissecting the anorectal canal from the vagina during a proctectomy, the space in the region anterolateral to the anorectal canal around the superior border of the anal canal should be approached before dissecting the median anterior region.

## Conflicts of interest

The authors have no conflicts of interest to declare.
